# Cross-Cultural Adaptation and Validation of the Norwich Patellar Instability (NPI) Score and the Banff Patellofemoral Instability Instrument (BPII) 2.0 in a Polish Pediatric Population

**DOI:** 10.3390/children12121708

**Published:** 2025-12-17

**Authors:** Alicja Fąfara, Jarosław Feluś, Kinga Żmijewska-Jasińska

**Affiliations:** 1 Institute of Physiotherapy, Faculty of Health Sciences, Jagiellonian University Medical College, 31-008 Kraków, Poland; 2 Children’s University Hospital in Kraków, 30-663 Kraków, Poland; jfelus@gmail.com; 3 Department of Orthopedics and Physiotherapy, Jagiellonian University Medical College, 31-008 Kraków, Poland; kinga.zmijewska@uj.edu.pl

**Keywords:** patellofemoral instability, functional assessment, COSMIN, disease-specific patient-reported outcome measure, cross-cultural adaptation, adolescent

## Abstract

**Highlights:**

**What are the main findings?**

- BPII 2.0 and NPI have been successfully translated into Polish and adopted in pediatric patients with patellofemoral instability.
- BPII 2.0 and NPI are valid and reliable disease-specific tools for functional assessment and quality of life measurement in patients with patellofemoral instability.

**What are the implications of the main findings?**

- Both questionnaires enable a comprehensive assessment, including Patient-Related Outcome Measures (PROMs).
- The Polish adaptation enables multi-center, international studies through uniform application of the functional assessment of patients with patellofemoral joint instability.

**Abstract:**

Introduction: Patellofemoral instability (PFI) is most prevalent in adolescents aged 10–17 years, yet disease-specific functional assessment tools validated for pediatric populations are limited. The Banff Patellofemoral Instability Instrument (BPII) 2.0 and the Norwich Patellar Instability (NPI) scores are disease-specific tools that have previously been validated in adults. The purpose of this study was to translate, culturally adapt, and validate the BPII 2.0 and NPI scores for Polish-speaking pediatric patients with PFI. Methods: The Polish versions of the BPII 2.0 and NPI were developed following Beaton’s cross-cultural adaptation guidelines. Patients aged 12–18 years with surgically treated recurrent patellofemoral joint instability completed the BPII 2.0, NPI, Anterior Knee Pain Scale (Kujala), Lysholm Knee Score, and Pedi-IKDC at a clinic visit and again 7–14 days later. The following psychometric properties were assessed: face validity, floor and ceiling effects, test–retest reliability (ICC), internal consistency (Cronbach’s α), and construct validity (Spearman Correlation Coefficients). Results: A total of 57 postoperative patients (19 males, 38 females; median age 16 years, range 12.25–18 years) participated 24–36 months after surgical stabilization. No floor or ceiling effects were observed. The test–retest reliability was excellent (ICC = 0.988 for BPII 2.0 (95% CI 0.977–0.994, *p* < 0.001); ICC = 0.997 for NPI (95% CI 0.995–0.998, *p* < 0.001)). Both instruments demonstrated excellent internal consistency (Cronbach’s α = 0.95 for BPII 2.0; α = 0.93 for NPI). The BPII 2.0 showed moderate to strong positive correlations with Lysholm (ρ = 0.69), Kujala (ρ = 0.69), and Pedi-IKDC (ρ = 0.57) and moderate negative correlation with NPI (ρ = −0.62), all of which were statistically significant (*p* < 0.001). Conclusion: The Polish versions of the BPII 2.0 and NPI scores demonstrated excellent reliability (ICC = 0.988 and 0.997, respectively), internal consistency (Cronbach’s α = 0.95 and 0.93, respectively), and construct validity in Polish-speaking adolescent patients with surgically treated recurrent patellofemoral instability. This is the first validation of the NPI in an exclusively pediatric population. These tools are suitable for clinical assessment and research in this specific population. Limitations include the postoperative-only cohort and absence of structural validity assessment.

## 1. Introduction

Patellar instability is a common orthopedic pathology in pediatric and adolescent populations [1,2,3]. Patellofemoral instability (PFI) may significantly limit physical activity, impair quality of life, and increase long-term risk of patellofemoral arthritis [4].

Comprehensive assessment of PFI requires validated functional assessment tools (NPI) [5,6] and health-related quality of life measurements (BPII 2.0) [7,8]. However, currently, no Polish-language disease-specific patient-reported outcome measures (PROMs) exist for assessing PFI in pediatric populations, limiting both clinical assessment capabilities and participation in international research.

Treatment of PFI includes both conservative and surgical approaches, with recurrence rates following initial dislocation ranging from 15% to 70% [9,10,11]. Patients with specific anatomical risk factors benefit from early surgical intervention to prevent recurrent instability [12,13,14,15,16].

The most frequently used knee assessment instruments either do not specifically address patellar instability (e.g., Kujala, Lysholm-Tegner, KOOS) or are not joint-specific (e.g., IKDC, Pedi-IKDC) [17,18]. Until recently, few disease-specific tools were available for assessing PFI. The Banff Patellofemoral Instability Instrument (BPII) 2.0 and the Norwich Patellar Instability (NPI) score are currently the only validated disease-specific PROMs for PFI [17].

The European Society of Sports Traumatology, Knee Surgery and Arthroscopy (ESSKA) Committee has endorsed these instruments as valid, reliable, disease-specific tools and recommends including them as a minimum standard in PFI studies [19]. The BPII 2.0 has been validated in adolescent populations [8] and translated into several languages, including Swedish [20], Spanish [21], German [22], Indonesian [23], Turkish [24], Norwegian [25], Arabic [26] and Dutch [27]. The NPI score has been validated primarily in adult populations [5,6,28,29] with translations into Turkish [24], Arabic [26], and Dutch [27]. Critically, the NPI score has not been validated exclusively in a pediatric population in any language, and neither instrument is available in Polish.

Therefore, the aims of this study were to (1) translate and culturally adapt the BPII 2.0 and NPI scores into Polish following standardized guidelines and (2) validate these instruments in Polish-speaking adolescent patients with surgically treated recurrent patellofemoral instability.

## 2. Materials and Methods

### 2.1. Ethical Approval and Permissions

The study was approved by the Ethics Committee of the Jagiellonian University Medical College (ID 1072.6120.213.2020 for BPII 2.0 and ID 1072.6120.214.2020 for NPI). Official recommendations for validation and Polish-language adaptation were obtained from the Banff Sport Medicine Foundation (for BPII 2.0), the original NPI author Dr. Toby Smith (Oxford University), and the Polish Arthroscopic Society (PTArtro). All participants and their legal guardians provided written informed consent.

### 2.2. Participants

Patients aged 12–18 years with clinically and radiologically confirmed recurrent patellofemoral joint instability who had undergone surgical stabilization were recruited from a tertiary pediatric orthopedic center between 2019 and 2022. All participants had been surgically treated for recurrent patellar instability (defined as at least two objective patellar dislocation episodes) or first-time acute patellar dislocation with non-comminuted osteochondral fracture >1 cm^2^. Surgical procedures included medial patellofemoral ligament reconstruction (MPFLr) alone or combined with tibial tuberosity osteotomy (TTO), trochleoplasty, or osteochondral fracture fixation.

The decision to include only surgically treated patients was made to (1) ensure a more homogeneous cohort for initial validation; (2) assess the instruments in patients with confirmed anatomical pathology requiring surgical intervention; and (3) evaluate the tools in a population where disease-specific outcome measurement is particularly important for monitoring surgical outcomes.

Patients were assessed 24–36 months postoperatively. This timeframe was selected to ensure patients were beyond the primary rehabilitation phase and had reached a relatively stable functional status, allowing for meaningful long-term assessment.

Inclusion criteria: 1. recurrent patellofemoral instability treated surgically; 2. age 12–18 years at assessment; 3. minimum 24 months post-surgical stabilization; 4. ability to understand and complete Polish-language questionnaires; 5. written informed consent from the patient and legal guardian.

Exclusion criteria: 1. additional lower extremity injuries or surgeries; 2. inability to understand or complete questionnaires; 3. withdrawal of consent.

Of 114 eligible patients identified, 19 declined participation and 38 were excluded due to additional lower extremity injuries or ending treatment after 18 years of age. No patients withdrew after enrollment.

### 2.3. Translation and Cross-Cultural Adaptation

The BPII 2.0 and NPI scores were translated and culturally adapted following Beaton’s guidelines [30]. The BPII 2.0 and NPI scores were translated and culturally adapted following Beaton’s guidelines [24]. Two independent translations were performed, followed by research team (AF, JF, KŻJ) review and back translations of the new Polish version of both questionnaires.

Face validity [31] was assessed in 15 patients with PFI (8 females, 7 males; median age 15 years, range 13–17 years; all with surgically treated recurrent patellar instability) who completed the preliminary Polish versions and provided feedback on comprehensibility and relevance. This sample was demographically representative of the intended validation population. No difficulties were reported, and no modifications were necessary. The final Polish versions were approved for validation testing (Supplement S1—Polish version of the BPII 2.0; Supplement S2—Polish version of the NPI score).

### 2.4. Validation Procedure

Eligible patients completed a standardized assessment protocol during a routine postoperative clinic visit, including (1) demographic and clinical data; (2) surgical procedure details; and (3) Patient-Reported Outcome Measures, including BPII 2.0, NPI, Anterior Knee Pain Scale (Kujala), Lysholm Knee Score, and Pediatric International Knee Documentation Committee Subjective Knee Evaluation Form (Pedi-IKDC). All patients completed questionnaires independently in a quiet clinic area with research staff available to answer procedural questions without influencing responses.

For test–retest reliability assessment, patients completed the BPII 2.0 and NPI again 7–14 days after the initial assessment. This interval was selected to minimize recall bias while ensuring clinical stability and representing a timeframe within which patients’ functional status would be expected to remain stable [32]. The second set of questionnaires was administered via email with instructions to complete them independently and return them within 48 h. Patients were asked to report any intervening events (e.g., trauma, new symptoms) that might affect responses; none were reported. We acknowledge that email-based administration may introduce potential measurement bias through uncontrolled completion conditions; however, the excellent ICC values obtained suggest this approach did not substantially compromise reliability measurement.

### 2.5. Psychometric Properties and a Priori Hypotheses

Sample size was determined according to COSMIN recommendations and Terwee et al. [31], suggesting a minimum of 50 patients for construct validity assessment. The following psychometric properties were evaluated:

Face validity: Assessed qualitatively during the pretesting phase to ensure questionnaire items were comprehensible and relevant to the target population.

Floor and ceiling effects: Considered present if >15% of respondents achieved the minimum or maximum possible score, respectively, following COSMIN guidelines [32].

Test–retest reliability: Assessed using intraclass correlation coefficient (ICC) with a two-way random effects model, absolute agreement (ICC_2,1_). ICC values ≥0.75 indicate excellent reliability [31].

Internal consistency: Evaluated using Cronbach’s alpha coefficient. Values between 0.70 and 0.95 are considered acceptable, with values >0.95 potentially indicating item redundancy [31].

Construct validity: Assessed by testing a priori hypotheses regarding correlations between BPII 2.0, NPI, and comparator PROMs using Spearman’s correlation coefficients.

A priori hypotheses for construct validity:

BPII 2.0 and NPI will show moderate to strong negative correlation (ρ = −0.50 to −0.70), as higher BPII 2.0 scores indicate better function while higher NPI scores indicate worse function.

BPII 2.0 will show moderate to strong positive correlations (ρ = 0.50 to 0.75) with Lysholm, Kujala, and Pedi-IKDC, as all measure knee function positively.

NPI will show moderate to strong negative correlations (ρ = −0.40 to −0.60) with Lysholm, Kujala, and Pedi-IKDC, as NPI measures dysfunction while these tools measure function.

Correlations will be stronger between disease-specific tools (BPII 2.0 and NPI) than between these and general knee assessment tools, demonstrating appropriate discriminant validity.

### 2.6. Statistical Analysis

Descriptive statistics included means, standard deviations, medians, and interquartile ranges. Data distribution was assessed using the Shapiro–Wilk test and visual inspection of histograms. Potential outliers were identified as values <Q1 −1.5 × IQR or >Q3 +1.5 × IQR.

Floor and ceiling effects were examined by calculating the percentage of participants achieving minimum or maximum scores. Test–retest reliability was assessed using ICC (two-way random effects, absolute agreement, single rater) with 95% confidence intervals. Internal consistency was evaluated using Cronbach’s alpha. Construct validity was assessed using Spearman’s rank correlation coefficients due to non-normal distribution of some variables. Statistical significance was set at *p* < 0.05. All analyses were performed using IBM SPSS Statistics Version 26.

## 3. Results

### 3.1. Demographics and Clinical Characteristics

Fifty-seven patients participated in the study (38 females, 19 males; median age 16 years, range 12.25–18 years). The operation was performed on the left side in 20 patients, the right in 31 patients, and bilaterally in 6 patients. The median time from surgery to assessment was 31 months (range 24–36 months). Surgical procedures included isolated MPFLr (n = 13), MPFLr + TTO (n = 24), MPFLr + trochleoplasty (n = 15), and MPFLr + osteochondral fracture fixation (n = 5). No missing data were recorded for any questionnaire items or demographic variables. The median interval between test and retest was 10 days (range 7–14 days). Demographic and clinical characteristics are presented in Table 1.

### 3.2. Descriptive Statistics

BPII 2.0 total scores ranged from 28.17 to 100.0 (mean 68.18 ± 20.14) at initial testing and 28.52 to 100.0 (mean 68.21 ± 20.08) at retest. NPI total scores ranged from 0.0 to 76.0 (mean 13.57 ± 16.01) at initial testing and 0.0 to 75.2 (mean 13.37 ± 15.71) at retest. No significant differences were observed between test and retest scores. Detailed descriptive statistics are shown in Table 2.

### 3.3. Floor and Ceiling Effects

The BPII 2.0 score distribution was slightly left-skewed, with most participants scoring near or above the mean. Only one participant (1.8%) achieved the maximum score of 100.0. No floor effect was observed.

The NPI score distribution was right-skewed, with predominantly low disability scores. Eight participants (14.0%) achieved the minimum score of 0.0. This falls below the 15% threshold; therefore, no floor or ceiling effects were present for either instrument.

### 3.4. Test–Retest Reliability

The test–retest reliability was excellent for both instruments. The ICC for BPII 2.0 was 0.988 (95% CI 0.977–0.994, *p* < 0.001). The ICC for NPI was 0.997 (95% CI 0.995–0.998, *p* < 0.001).

### 3.5. Internal Consistency

Both instruments demonstrated excellent internal consistency. Cronbach’s alpha for BPII 2.0 was 0.95, and for NPI, it was 0.93, both of which are within the acceptable range and do not indicate item redundancy.

### 3.6. Construct Validity

All a priori hypotheses were confirmed. BPII 2.0 and NPI demonstrated a moderate negative correlation (ρ = −0.62, *p* < 0.001), as hypothesized. BPII 2.0 showed strong positive correlations with Lysholm (ρ = 0.69, *p* < 0.001) and Kujala (ρ = 0.69, *p* < 0.001) and moderate positive correlation with Pedi-IKDC (ρ = 0.57, *p* < 0.001). NPI showed moderate negative correlations with Lysholm (ρ = −0.56, *p* < 0.001), Kujala (ρ = −0.51, *p* < 0.001), and Pedi-IKDC (ρ = −0.53, *p* < 0.001). All correlations were statistically significant and aligned with predicted directions and magnitudes. Detailed correlation results are presented in Table 3. All a priori hypotheses were confirmed, with BPII 2.0 and NPI showing the expected moderate to strong correlations in predicted directions with established knee assessment tools.

## 4. Discussion

This study demonstrates that the Polish versions of the BPII 2.0 and NPI scores are reliable, internally consistent, and valid tools for assessing Polish-speaking adolescent patients with surgically treated recurrent patellofemoral instability. To our knowledge, this is the first validation of the NPI score exclusively in a pediatric population, expanding the evidence base for this instrument beyond its original adult validation. Additionally, this is the first availability of disease-specific PFI assessment tools in Polish.

The excellent test–retest reliability (ICC = 0.988, 95% CI 0.977–0.994 for BPII 2.0; ICC = 0.997, 95% CI 0.995–0.998 for NPI) confirms measurement stability and surpasses or equals values reported in other language validations [16,17,18,20,21,23]. The high internal consistency (Cronbach’s α = 0.95 for BPII 2.0; α = 0.93 for NPI) demonstrates strong inter-item correlation, aligning with the original instrument development [14,16] and other adaptations [19,20,21,23,24,25,26]. While Cronbach’s alpha values >0.90 can sometimes suggest item redundancy, values in the 0.90–0.95 range are generally considered acceptable for clinical outcome measures and do not necessarily indicate problematic redundancy [31]. Both values in our study fall within this acceptable range. Future research could explore potential item redundancy through factor analysis or item response theory, though this was beyond the scope of the current validation study.

All a priori hypotheses for construct validity were confirmed. The moderate negative correlation between BPII 2.0 and NPI (ρ = −0.62) is expected given their opposite scoring directions and demonstrates appropriate convergent validity. The moderate to strong correlations with Lysholm and Kujala (ρ = 0.51 to 0.69) support construct validity while remaining sufficiently distinct to justify disease-specific instruments.

The moderate correlations with Pedi-IKDC (ρ = 0.57 for BPII 2.0; ρ = −0.53 for NPI) appropriately demonstrate discriminant validity. These slightly lower correlations compared to Lysholm and Kujala likely reflect the fact that Pedi-IKDC assesses general knee function rather than instability-specific concerns. This pattern suggests that while BPII 2.0 and NPI measure constructs related to overall knee function, they capture distinct aspects specific to patellar instability that are not fully addressed by general knee assessment tools.

The correlation patterns suggest that, while BPII 2.0 and NPI measure overlapping constructs related to patellar instability, they capture complementary aspects. The BPII 2.0 emphasizes functional limitations and quality of life impact, while the NPI focuses more specifically on instability symptoms and episodes. This complementarity supports using both instruments in comprehensive assessment, as recommended by ESSKA guidelines [15].

No floor or ceiling effects were observed, indicating that the instruments can detect change across the full spectrum of dysfunction in postoperative adolescent patients. The slight left-skew of BPII 2.0 scores and right-skew of NPI scores are expected in a postoperative cohort 2–3 years after surgery, reflecting generally good outcomes while maintaining sensitivity to residual symptoms.

### 4.1. Study Limitations

Several limitations warrant consideration. First, the study population consisted exclusively of surgically treated pediatric patients assessed postoperatively, along with the absence of asymptomatic controls. Generalizability to conservatively managed patients, younger children, preoperative assessment, or comparison with healthy controls is unknown. Future research should validate these instruments in diverse PFI populations, including nonoperative management, broader age ranges, and preoperative settings.

Second, structural validity was not assessed due to sample size constraints. While both instruments have established single-factor structures in their original populations [6,7,8], confirmatory factor analysis in larger Polish samples would strengthen validation evidence and confirm measurement equivalence across cultures.

Third, retest questionnaires were administered via email rather than in person, which may introduce measurement bias through uncontrolled completion conditions. However, no intervening events were reported, and the excellent ICC values (both >0.98) suggest that this approach did not substantially compromise reliability measurement.

Fourth, the relatively homogeneous surgical population and single-center recruitment limit generalizability. Multi-center studies including diverse treatment approaches and geographic regions would provide more robust validation evidence.

Fifth, this study employed Classical Test Theory (CTT) methods rather than Modern Test Theory (MTT) approaches such as Rasch analysis or Item Response Theory. While MTT methods provide valuable additional information about measurement properties, including item difficulty hierarchies, person ability estimates, and differential item functioning across subgroups [33,34], we prioritized CTT methods for several reasons. First, both the BPII 2.0 and NPI were originally developed using CTT approaches [6,7], and all subsequent international validations have maintained this methodological consistency to enable cross-cultural comparability [19,20,21,22,23,24,25,26]. Second, our primary aim was cross-cultural adaptation of existing instruments rather than de novo scale development. COSMIN guidelines for cross-cultural adaptation emphasize demonstrating measurement equivalence through CTT properties—specifically reliability, internal consistency, and construct validity [31,32]—which directly address whether the translated instruments maintain the measurement characteristics of the original versions. Third, our sample size (N = 57), while adequate for CTT validation per COSMIN recommendations [31], is insufficient for robust Rasch analysis. Stable item parameter estimation in Rasch models typically requires sample sizes of 150–250 or larger, with even larger samples needed when examining differential item functioning across multiple subgroups [34,35]. Our relatively small subgroups (males n = 19, females n = 38; four surgical procedure types ranging from n = 5 to n = 24) would preclude reliable DIF analysis. Fourth, both instruments have well-established unidimensional structures confirmed through multiple international validations [17,20,21,22,23,24,25,26,27], and our excellent reliability (ICC > 0.98), internal consistency (α = 0.93–0.95), and construct validity results demonstrate successful maintenance of these properties in the Polish adaptation. Finally, maintaining CTT-based validation ensures clinical interpretability consistent with established scoring systems and enables direct comparison with international outcome data, supporting the practical utility of these instruments in routine clinical practice. Future research with larger, diverse Polish samples should employ MTT approaches to further examine item-level properties, potential item redundancy, measurement invariance across demographic and clinical subgroups, and item difficulty calibration. Such analyses would provide complementary evidence strengthening the psychometric foundation of these instruments in Polish-speaking populations and could inform potential scale refinements if needed.

Sixth, responsiveness to change over time was not assessed. Future longitudinal studies should evaluate the instruments’ ability to detect clinically meaningful changes following treatment interventions.

### 4.2. Clinical Implications

The availability of validated Polish versions of BPII 2.0 and NPI enables standardized, disease-specific functional assessment of Polish-speaking pediatric patients with PFI in routine pediatric orthopedic practice. These tools can be used for (1) baseline assessment to guide treatment decisions between conservative and surgical management; (2) monitoring patient progress during rehabilitation; (3) evaluating surgical outcomes at follow-up; (4) identifying patients with residual symptoms requiring additional intervention; and (5) facilitating shared decision-making through structured outcome measurement.

Polish orthopedic surgeons and physiotherapists can now assess baseline functional status and treatment effects using internationally recognized, validated instruments, enabling participation in multi-center international studies and meaningful cross-cultural outcome comparisons. The validation of NPI in an exclusively pediatric population provides new evidence supporting its use in adolescents, expanding the tool’s applicability beyond its original adult validation. Combined use of BPII 2.0 and NPI, as recommended by ESSKA [15], provides comprehensive assessment capturing both functional impact and instability-specific symptoms.

## 5. Conclusions

The Polish versions of the BPII 2.0 and NPI scores demonstrated excellent test–retest reliability, internal consistency, and construct validity in Polish-speaking pediatric patients with surgically treated recurrent patellofemoral instability. This represents the first validation of the NPI score exclusively in a pediatric population. These findings support their use as reliable and valid disease-specific outcome measures in this specific population.

Generalizability is limited to surgically treated pediatric patients in the postoperative phase. Future research should include structural validity assessment through confirmatory factor analysis, validation in conservatively managed and preoperative patients, broader age ranges including younger children, inclusion of asymptomatic controls, responsiveness evaluation, and Modern Test Theory approaches with larger samples to examine item-level properties and measurement invariance, to further establish these instruments’ measurement properties across diverse Polish-speaking pediatric populations with patellofemoral instability.

## Figures and Tables

**Table 1:** Demographic and clinical characteristics of study participants (N = 57).

Parameter	Value
Sex, n (%)	
Female	38 (66.7)
Male	19 (33.3)
Age (years)	Median 16 (range 12.25–18.0)
Operated side, n (%)	
Left	20 (35.1)
Right	31 (54.4)
Bilateral	6 (10.5)
Time since surgery (months)	Median 31 (range 24–36)
Surgical procedure, n (%)	
Isolated MPFLr	13 (22.8)
MPFLr + TTO	24 (42.1)
MPFLr + trochleoplasty	15 (26.3)
MPFLr + OCF fixation	5 (8.8)

**Table 2:** Descriptive Statistics for BPII 2.0 and NPI Scores at Test and Retest (N = 57). Distribution of BPII 2.0 and NPI scores showing excellent test–retest stability with nearly identical means and standard deviations across administrations.

Instrument	N	Mean	SD	Median	IQR	Min	Max	Range
BPII 2.0 (test)	57	68.18	20.14	69.95	52.43–85.56	28.17	100.0	71.83
BPII 2.0 (retest)	57	68.21	20.08	69.95	52.52–84.70	28.52	100.0	71.48
NPI (test)	57	13.57	16.01	7.20	2.40–18.80	0.0	76.0	76.0
NPI (retest)	57	13.37	15.71	7.90	2.40–19.42	0.0	75.2	75.2

SD = standard deviation; IQR = interquartile range (25th–75th percentile); Min = minimum; Max = maximum. Higher BPII 2.0 scores indicate better function; higher NPI scores indicate greater disability.

**Table 3:** Construct validity: Spearman correlation coefficients between BPII 2.0, NPI, and comparator instruments.

Comparison	Spearman ρ	95% CI	*p*-Value	Interpretation
BPII 2.0 vs. NPI	−0.62	−0.75 to −0.45	<0.001	Moderate negative (convergent validity)
BPII 2.0 vs. Lysholm	0.69	0.54 to 0.80	<0.001	Strong positive
BPII 2.0 vs. Kujala	0.69	0.54 to 0.80	<0.001	Strong positive
BPII 2.0 vs. Pedi-IKDC	0.57	0.38 to 0.72	<0.001	Moderate positive
NPI vs. Lysholm	−0.56	−0.71 to −0.36	<0.001	Moderate negative
NPI vs. Kujala	−0.51	−0.67 to −0.30	<0.001	Moderate negative
NPI vs. Pedi-IKDC	−0.53	−0.69 to −0.32	<0.001	Moderate negative

CI = confidence interval. Correlation strength interpretation: 0.00–0.30 weak, 0.30–0.70 moderate, 0.70–1.00 strong.

## Data Availability

The raw data supporting the conclusions of this article will be made available by the authors on request.

## Supplementary Materials

The following supporting information can be downloaded at: https://www.mdpi.com/article/10.3390/children12121708/s1: Polish pediatric version of the BPII 2.0 and NPI.

## Abbreviations

The following abbreviations are used in this manuscript:

BPII 2.0	Banff Patellofemoral Instability Instrument
NPI	Norwich Patellar Instability Score
PROMs	Patient-Related Outcome Measures
PFJ	Patellofemoral joint
PFI	Patellofemoral instability
KOOS	Knee Injury and Osteoarthritis Outcome Score
IKDC	International Knee Documentation Committee
Pedi-IKDC	Pediatric International Knee Documentation Committee
COSMIN	COnsensus-based Standards for the selection of health Measurement INstruments
MPFLr	Medial patellofemoral ligament reconstruction
OCF	Osteochondral fracture fixation
TTO	Tibial tuberosity osteotomy
CTT	Classical Test Theory
MTT	Modern Test Theory
