# Supplementary material for: Cross-Cultural Adaptation and Validation of the Norwich Patellar Instability (NPI) Score and the Banff Patellofemoral Instability Instrument (BPII) 2.0 in a Polish Pediatric Population
Source: Children (Basel). 2025 Dec 17;12(12):1708. doi: 10.3390/children12121708 (PMC12732070; doi:10.3390/children12121708)
Supplement: Supplementary file 1 [file children-12-01708-s001.zip › children-3995253-supplementary.pdf]

S1. BANFF PATELLOFEMORAL INSTABILITY INSTRUMENT (BPII) 2.0

NARZĘDZIE OCENY NIESTABILNOŚCI STAWU RZEPKOWO-UDOWEGO BANFF 2.0

WERSJA POLSKA	ENGLISH VERSION
Imię i Nazwisko: ___________________________________	Patient Name: ___________________________________
Data: ___________________________________	Date: ___________________________________
**INSTRUKCJA:**	**INSTRUCTIONS:**
Proszę odpowiedzieć na każde pytanie w odniesieniu do aktualnego stanu kolana. Na linii poniżej proszę zaznaczyć ukośnikiem (/) miejsce odpowiadające Twojej odpowiedzi (0-100).	Please answer each question with respect to your current knee condition. On the line below, mark with a slash (/) the point corresponding to your answer (0-100).
**CZĘŚĆ A: OBJAWY I DOLEGLIWOŚCI FIZYCZNE**	**PART A: SYMPTOMS AND PHYSICAL COMPLAINTS**
**1. Jak bardzo dokuczliwe/uciążliwe są dla Ciebie dolegliwości, które powodują 'wyskakiwanie' lub niestabilność rzepki?**<br>0 ____________________________________ 100<br>Niezwykle uciążliwe W ogóle nie jest uciążliwe	**1. How troubled are you by "popping out" or instability of your kneecap?**<br>0 ____________________________________ 100<br>Extremely troubled Not troubled at all
**2. Jak duży ból w kolanie lub dyskomfort czujesz podczas jakiejkolwiek dłuższej aktywności (powyżej pół godziny)?**<br>0 ____________________________________ 100<br>Silny ból W ogóle bez bólu	**2. How much pain or discomfort do you get in your knee with any king of prolonged activity (greater than half an hour)? For example: standing, walking, sport, etc.**<br>0 ____________________________________ 100<br>Severe pain No pain at all
**3. Jak duży ból w kolanie lub dyskomfort czujesz podczas dłuższego siedzenia (powyżej pół godziny)?**<br>0 ____________________________________ 100<br>Silny ból W ogóle bez bólu	**3. How much pain or discomfort do you get in your knee during prolonged sitting (greater than half an hour)?**<br>0 ____________________________________ 100<br>Severe pain No pain at all
**4. Czy masz ograniczenia ruchomości kolana?**<br>0 ____________________________________ 100<br>Olbrzymie ograniczenia Pełny ruch w kolanie	**4. Do you have any loss of motion of your knee?**<br>0 ____________________________________ 100<br>Severe limitations No loss of motion
**5. Jak bardzo osłabione jest Twoje kolano?**<br>0 ____________________________________ 100<br>Ekstremalnie słabe Nie czuję, że jest słabe	**5. How weak does your knee feel?**<br>0 ____________________________________ 100<br>Extremely weak Not weak at all
**CZĘŚĆ B: KWESTIE ZWIĄZANE Z PRACĄ/SZKOŁĄ**	**PART B: WORK AND/OR SCHOOL RELATED CONCERNS**
**6. Jaką trudność w szkole/pracy sprawiają Ci ruchy skrętne lub rotacyjne?**<br>0 ____________________________________ 100<br>Ekstremalne trudności W ogóle nie sprawia trudności	**6. How much difficulty do you have because of your knee with turning or pivoting motion at school/work?**<br>0 ____________________________________ 100<br>Extreme difficulty No difficulty at all
**7. Jaką trudność w szkole/pracy sprawia Ci kolano podczas czynności wymagających przykucnięcia?**	**7. How much difficulty do you have with squatting at school/work?**<br>0 ____________________________________ 100

0 ____________________________________ 100<br>Ekstremalne trudności W ogóle nie sprawia trudności	Extreme difficulty No difficulty at all
**8. Jak bardzo martwisz się, że nie jesteś w szkole i/lub w pracy z powodu problemów z kolanem?**<br>0 ____________________________________ 100<br>Bardzo mnie martwi W ogóle mnie nie martwi	**8. How much of a concern is it for you to miss time from work/school because of knee problems?**<br>0 ____________________________________ 100<br>Extreme concern No concern at all
**9. Czy problemy związane z urazem kolana spowodowały trudności finansowe dla Ciebie lub/i Twojej rodziny?**<br>0 ____________________________________ 100<br>Olbrzymie trudności finansowe Nie mam trudności finansowych	**9. Has the cost of your knee injury created financial hardship for you or for your family?**<br>0 ____________________________________ 100<br>Severe financial hardship No financial hardship at all
**CZĘŚĆ C: REKREACJA/SPORT/AKTYWNOŚĆ**	**PART C: RECREATION/SPORT/ACTIVITY**
**10. Jak bardzo obawiasz się, że rekreacyjne uprawianie sportu może pogorszyć stan Twojego kolana?**<br>0 ____________________________________ 100<br>Bardzo się obawiam Nie mam takich obaw	**10. Howconcerned are you that recreational and/or sports activities make your knee worse?**<br>0 ____________________________________ 100<br>Extremely concerned Not concerned at all
**11. Czy musisz zachowywać ostrożność podczas uprawiania rekreacyjnych sportów?**<br>0 ____________________________________ 100<br>Zawsze ostrożnie Nie muszę zachowywać ostrożności	**11. Do you have participate in recreational and/or sports activities with caution?**<br>0 ____________________________________ 100<br>Always with caution Never with caution
**12. Jak bardzo obawiasz się 'wyskakiwania' rzepki podczas uprawiania sportu lub aktywności rekreacyjnych?**<br>0 ____________________________________ 100<br>Bardzo się obawiam Nie mam takich obaw	**12. How fearful of your knee 'popping out' when participating in recreational and/or sports activities?**<br>0 ____________________________________ 100<br>Extremely fearful No fearful at all
**13. Jak bardzo obawiasz się chodzenia po nierównym podłożu, mokrej nawierzchni lub po lodzie?**<br>0 ____________________________________ 100<br>Bardzo się obawiam Nie mam takich obaw	**13. How concerned are you when walking on uneven ground, on wet surface or walking on ice?**<br>0 ____________________________________ 100<br>Extremely concerned No concerned at all
**14. Czy jesteś w stanie w pełni zaangażować się w aktywność sportową/rekreacyjną na miarę swoich pełnych możliwości?**<br>0 ____________________________________ 100<br>W ogóle nie jestem w stanie Zawsze jestem w stanie	**14. Are you able to give your full effort in your sports/recreational activity?**<br>0 ____________________________________ 100<br>Not able at all Always able
**CZĘŚĆ D: ŻYCIE CODZIENNE, JAKOŚĆ ŻYCIA**	**PART D: LIFESTYLE**
**15. Jak silne są Twoje ogólne obawy dotyczące bezpieczeństwa ze względu na problem z kolanem?**<br>0 ____________________________________ 100<br>Silne obawy Nie mam obaw	**15. How concerned are you with general safety issues because of your knee problems?**<br>0 ____________________________________ 100<br>Extremely concerned No concerned at all
**16. W jakim stopniu problem z kolanem ograniczył możliwość dbania o sprawność fizyczną i możliwość jej utrzymania?**<br>0 ____________________________________ 100<br>Bardzo mnie ograniczył W ogóle mnie nie ograniczył	**16. How much has your ability to exercise and maintain fitness been limited by your knee problems?**<br>0 ____________________________________ 100<br>Totally limited Not limited at all
**17. Jak bardzo problem z kolanem ograniczył Twoją możliwość cieszenia się z życia?**<br>0 ____________________________________ 100	**17. How much has your enjoyment of life been limited by your knee problems?**<br>0 ____________________________________ 100

Bardzo mnie ograniczył W ogóle mnie nie ograniczył	Totally limited Not limited at all
**18. Czy unikasz jakichkolwiek aktywności towarzyskich z rodziną oraz/lub przyjaciółmi ze względu na problemy z kolanem?**<br>0 ______________________________________ 100<br>Zawsze unikam Nigdy nie unikam	**18. Do you avoid lifestyle activities with family and/or friends due to knee problems?**<br>0 ______________________________________ 100<br>Always avoid Never avoid
**19. Czy ze względu na problemy z kolanem musisz planować swoją aktywność życiową lub towarzyską w znacznie bardziej dokładnym stopniu?**<br>0 ______________________________________ 100<br>Zawsze muszę planować Nigdy nie muszę planować	**19. Do you have to plan out your lifestyle and social activities more than your family and/or friends because of your knee problems?**<br>0 ______________________________________ 100<br>Always have to plan Never have to plan
**CZĘŚĆ E: KWESTIE SPOŁECZNE I EMOCJONALNE**	**PART E: SOCIAL AND EMOTIONAL ISSUES**
**20. Czy ze względu na problemy z kolanem czujesz się sfrustrowany/a, że Twoje potrzeby rekreacyjne lub sportowe nie mogą być zaspokojone?**<br>0 ______________________________________ 100<br>Niezwykle sfrustrowany/a Nie jestem tym w ogóle sfrustrowany	**20. Are you feel frustrated that your recreational or competitive needs are no longer being met because of your knee problems?**<br>0 ______________________________________ 100<br>Extremely frustrated Not frustrated at all
**21. Czy miałeś/aś trudności, żeby poradzić sobie emocjonalnie z problemami z kolanem?**<br>0 ______________________________________ 100<br>Tak, ogromne trudności W ogóle nie miałem z tym trudności	**21. Have you had difficulty to emotionally cope with your knee problems?**<br>0 ______________________________________ 100<br>Extreme difficulties No difficulties at all
**22. Jak często jesteś zirytowany problemami z kolanem?**<br>0 ______________________________________ 100<br>Cały czas Nigdy	**22. How often are you nervous about your knee?**<br>0 ______________________________________ 100<br>Always nervous Never
**23. Jak bardzo obawiasz się kolejnego urazu kolana?**<br>0 ______________________________________ 100<br>Zawsze się obawiam W ogóle się nie obawiam	**23. How fearful are you of re-injuring your knee?**<br>0 ______________________________________ 100<br>Extremely fearful Not fearful at all

S2. NORWICH PATELLAR INSTABILITY (NPI) SCORE

**WERSJA POLSKA**	**ENGLISH VERSION**
Imię i Nazwisko: _________________________	Patient Name: _________________________
Data: _________________________	Date: _________________________
**INSTRUKCJA:**	**INSTRUCTIONS:**
Poniżej znajduje się lista czynności, które mogą powodować w stawie kolanowym odczucie, że Twoja rzepka zaraz wypadnie ze stawu lub że jest niestabilna. Proszę zaznaczyć pole, które najlepiej opisuje jak często masz to odczucie podczas każdej aktywności.	Below is a list of activities that may cause you to feel that your kneecap is about to slip out or is unstable. Please mark the box that best describes how often you have this feeling during each activity.
1. Skręcanie/zmiana kierunku w czasie gry/sportu	1. Twisting/turning during sports/games
Zawsze □ Często □ Czasem □ Rzadko □ Nigdy □ Nie robię □	Always □ Often □ Sometimes □ Rarely □ Never □ Don't do □
2. Zmiana kierunku w czasie biegania	2. Changing direction whilst running
Zawsze □ Często □ Czasem □ Rzadko □ Nigdy □ Nie robię □	Always □ Often □ Sometimes □ Rarely □ Never □ Don't do □
3. Bieganie po linii prostej, po nierównej powierzchni	3. Running in a straight line on uneven surface
Zawsze □ Często □ Czasem □ Rzadko □ Nigdy □ Nie robię □	Always □ Often □ Sometimes □ Rarely □ Never □ Don't do □
4. Chód po śliskiej, mokrej lub oblodzonej powierzchni	4. Walking on slippery, wet or icy surface
Zawsze □ Często □ Czasem □ Rzadko □ Nigdy □ Nie robię □	Always □ Often □ Sometimes □ Rarely □ Never □ Don't do □
5. Bieg bokiem	5. Running sideways
Zawsze □ Często □ Czasem □ Rzadko □ Nigdy □ Nie robię □	Always □ Often □ Sometimes □ Rarely □ Never □ Don't do □
6. Skakanie na jednej nodze	6. Hopping on one leg
Zawsze □ Często □ Czasem □ Rzadko □ Nigdy □ Nie robię □	Always □ Often □ Sometimes □ Rarely □ Never □ Don't do □
7. Podskoki	7. Jumping
Zawsze □ Często □ Czasem □ Rzadko □ Nigdy □ Nie robię □	Always □ Often □ Sometimes □ Rarely □ Never □ Don't do □
8. Bieg – na wprost po równym podłożu	8. Running straight ahead on even ground
Zawsze □ Często □ Czasem □ Rzadko □ Nigdy □ Nie robię □	Always □ Often □ Sometimes □ Rarely □ Never □ Don't do □
9. Schodzenie ze schodów	9. Going down stairs
Zawsze □ Często □ Czasem □ Rzadko □ Nigdy □ Nie robię □	Always □ Often □ Sometimes □ Rarely □ Never □ Don't do □
10. Kucanie	10. Squatting
Zawsze □ Często □ Czasem □ Rzadko □ Nigdy □ Nie robię □	Always □ Often □ Sometimes □ Rarely □ Never □ Don't do □
11. Klękanie	11. Kneeling
Zawsze □ Często □ Czasem □ Rzadko □ Nigdy □ Nie robię □	Always □ Often □ Sometimes □ Rarely □ Never □ Don't do □
12. Chodzenie na wprost po nierównym podłożu	12. Walking straight ahead on uneven ground
Zawsze □ Często □ Czasem □ Rzadko □ Nigdy □ Nie robię □	Always □ Often □ Sometimes □ Rarely □ Never □ Don't do □
13. Wchodzenie po schodach	13. Going up stairs
Zawsze □ Często □ Czasem □ Rzadko □ Nigdy □ Nie robię □	Always □ Often □ Sometimes □ Rarely □ Never □ Don't do □
14. Wchodzenie na/przekraczanie dużego stopnia	14. Stepping up/over a large step
Zawsze □ Często □ Czasem □ Rzadko □ Nigdy □ Nie robię □	Always □ Often □ Sometimes □ Rarely □ Never □ Don't do □
15. Krzyżowanie nóg podczas siedzenia	15. Crossing legs whilst sitting
Zawsze □ Często □ Czasem □ Rzadko □ Nigdy □ Nie robię □	Always □ Often □ Sometimes □ Rarely □ Never □ Don't do □

16. Chodzenie na wprost po równym podłożu<br>Zawsze □ Często □ Czasem □ Rzadko □ Nigdy □ Nie robię □	16. Walking straight ahead on even ground<br>Always □ Often □ Sometimes □ Rarely □ Never □ Don't do □
17. Wsiadanie/wysiadanie z samochodu<br>Zawsze □ Często □ Czasem □ Rzadko □ Nigdy □ Nie robię □	17. Getting in/out of car<br>Always □ Often □ Sometimes □ Rarely □ Never □ Don't do □
18. Zakręcanie ciężkim wózkiem w supermarkecie<br>Zawsze □ Często □ Czasem □ Rzadko □ Nigdy □ Nie robię □	18. Turning with a heavy shopping trolley<br>Always □ Often □ Sometimes □ Rarely □ Never □ Don't do □
19. Obracanie się przez ramię, aby spojrzeć w tył<br>Zawsze □ Często □ Czasem □ Rzadko □ Nigdy □ Nie robię □	19. Turning to look behind over shoulder<br>Always □ Often □ Sometimes □ Rarely □ Never □ Don't do □
